# Safety, tolerability, pharmacokinetics, and pharmacodynamics of a long-acting release (LAR) formulation of pasireotide (SOM230) in patients with gastroenteropancreatic neuroendocrine tumors: results from a randomized, multicenter, open-label, phase I study

**DOI:** 10.1007/s00280-013-2202-1

**Published:** 2013-06-14

**Authors:** Edward M. Wolin, Ke Hu, Gareth Hughes, Emmanuel Bouillaud, Vanessa Giannone, Karina Hermosillo Resendiz

**Affiliations:** 1 Carcinoid/Neuroendocrine Tumor Program, Samuel Oschin Cancer Center, Cedars-Sinai Medical Center, 8700 Beverly Boulevard, Los Angeles, CA 90048 USA; 2 Novartis Oncology, One Health Plaza, East Hanover, NJ 07936 USA; 3 Novartis Pharma AG, 4002 Basel, Switzerland

**Keywords:** Pasireotide (SOM230), Gastroenteropancreatic neuroendocrine tumor (GEP NET), Safety, Pharmacokinetics, Pharmacodynamics

## Abstract

**Purpose:**

Pasireotide (SOM230), a novel multireceptor ligand somatostatin analog (SSA), binds with high affinity to four of the five somatostatin receptor subtypes (sst_1–3, 5_). This study evaluated the safety, tolerability, pharmacokinetics, and pharmacodynamics profiles of pasireotide long-acting release (LAR) formulation in patients with advanced gastroenteropancreatic neuroendocrine tumor (GEP NET) refractory to other SSAs.

**Methods:**

In this randomized, multicenter, open-label, phase II study, patients with biopsy-proven primary or metastatic GEP NET refractory to available SSAs were randomly assigned 1:1:1 to receive pasireotide LAR by deep intragluteal injection at a dose of 20, 40, or 60 mg once every 28 days for 3 months.

**Results:**

Forty-two patients received pasireotide LAR. Adverse events were reported by 34 (81 %) patients, with the most frequently reported including diarrhea, fatigue, abdominal pain, and nausea. Mean fasting glucose levels were increased compared with baseline at all points throughout the study. After the third injection of pasireotide LAR, the median trough plasma concentrations on day 84 were 4.82, 12.0, and 19.7 ng/mL in the 20-, 40-, and 60-mg treatment groups, respectively. Drug accumulation was limited for each dose based on the increase in trough concentrations after the first to third injections (accumulation ratios were approximately 1 from all dose levels).

**Conclusions:**

This study demonstrated that a new, once-monthly, intramuscular LAR formulation of pasireotide was well tolerated in patients with advanced GEP NET. Steady state levels of plasma pasireotide were achieved after three injections.

## Introduction

Gastroenteropancreatic neuroendocrine tumors (GEP NET) occur at an annual incidence of approximately 5 cases per 100,000 per year, and the estimated prevalence is >100,000 persons in the United States [1, 2]. Thus, these tumors are significantly more prevalent than most other gastrointestinal malignancies [2]. Patients with GEP NET have a 5-year survival rate of 67.5 % across all tumor types. Most patients present with distant metastases; the 5-year survival rate for this population is 40.9 % [1]. GEP NET can secrete a wide range of biologically active amines and peptides. The most common is serotonin, which is responsible for the classic symptoms of carcinoid syndrome (diarrhea, flushing, bronchoconstriction, and right-sided valvular heart disease) [3].

Somatostatin inhibits hormone release and cell growth through binding to specific, cell surface, G-protein–coupled receptors, of which five distinct subtypes (sst_1–5_) have been characterized [4–6]. In pancreatic and gastrointestinal endocrine tumors, sst_2_ expression predominates, although multiple other subtypes have also been found [7]. The limited clinical use of native somatostatin, because of its very short half-life (<3 min) and the impact of rebound hypersecretion, has necessitated the development of more clinically useful analogs [6, 8, 9]. Development of the somatostatin analog (SSA) octreotide (Sandostatin; Novartis) was reported in 1982 [10] and was followed by advancement of several other cyclic octapeptides, all of which demonstrated increased resistance to peptidase inactivation, substantially longer half-lives, and improved pharmacologic efficacy [8]. Unlike natural somatostatin, octreotide binds with high affinity only to the sst_2_ receptor subtype and with lower binding affinity to the sst_5_ receptor, and its activity does not cause rebound hormonal hypersecretion [7, 11].

Pasireotide (SOM230) is a novel, multireceptor ligand SSA that binds with high affinity to four of the five somatostatin receptor subtypes (sst_1–3,5_) [12]. The unique binding profile of this SSA makes pasireotide a promising new therapy for patients with advanced GEP NET, including those refractory or resistant to octreotide and lanreotide. In clinical trials, pasireotide subcutaneous (SC) formulation has demonstrated efficacy and safety in patients with acromegaly and Cushing’s disease [13–15]. In addition, SC injection of pasireotide effectively reduces the symptoms of diarrhea and flushing in patients with metastatic NET refractory or resistant to octreotide long-acting repeatable (LAR) [16]. In this phase II study, complete or partial symptom control was achieved in 27 % of patients, and 57 % of patients had stable disease at 6 months. Adverse events (AEs), the most common of which were gastrointestinal, were consistent with other SSAs. Increases in blood glucose were also reported, but these were generally well controlled and rarely led to premature discontinuation of pasireotide. Although the SC formulation of pasireotide required a twice-daily administration schedule, a recent study [17] showed that continuous 7-day infusion of pasireotide in healthy volunteers was safe and well tolerated, presenting the opportunity for development of an LAR formulation. This formulation is administered intramuscularly (IM) monthly (once every 28 days). Early clinical data suggest a favorable safety profile in combination with everolimus in patients with advanced GEP NET [18].

The primary objective of the present study was to evaluate the safety/tolerability and pharmacokinetic (PK) profiles of monthly doses of pasireotide LAR (20, 40, and 60 mg/month) in patients with advanced GEP NET. The secondary objectives of this study included an exploratory pharmacodynamic (PD) assessment of the effect of pasireotide LAR on bowel movement frequency—an important efficacy endpoint of symptom control in patients with GEP NET who have carcinoid syndrome and intractable diarrhea.

## Methods

### Study design

This was a randomized, multicenter, open-label, phase I study. After screening, eligible patients were randomly assigned on a 1:1:1 basis to receive pasireotide LAR IM, by deep intragluteal injection, at a dose of 20, 40, or 60 mg, once every 28 days for 3 months.

Patients who had received recent therapy with an SSA were required to complete a washout period before baseline assessment. The washout period was 8 weeks for patients who received octreotide LAR, lanreotide autogel, or any other long-acting SSA; 4 weeks for those who received lanreotide SR; and 2 days for those who received subcutaneous octreotide, subcutaneous pasireotide, or another short-acting SSA. Further use of these drugs was not permitted for the duration of the study. Before baseline, each patient naive to subcutaneous pasireotide received a single dose of subcutaneous pasireotide (300 μg) and was observed for ≥5 days to ensure adequate pasireotide tolerability. Subcutaneous dosing was not required for patients who had previously received subcutaneous pasireotide outside this study.

After overnight fast, all patients underwent baseline measurements of blood glucose, insulin, and glucagon, followed by a standardized breakfast containing ≤100 g carbohydrate. The first dose of pasireotide LAR was administered at about 8:00 AM on the first day of dosing, and subsequent doses were administered as close as possible to that time of day. Patients were assessed weekly for the first 28 days of the study and every 2 weeks thereafter. Blood samples for pharmacokinetic analyses and for evaluation of blood glucose, insulin, and glucagon levels were obtained at all assessments. Vital signs, blood chemistry, complete blood count (CBC), and electrocardiogram (ECG) were evaluated throughout the study and at study completion. Bowel movements were recorded throughout the study using a daily diary. After the core 3-month treatment phase and at the discretion of the investigator, an extension treatment phase of 3 months was possible and could be followed by a second extension treatment phase.

Patients were to be discontinued from the study if they experienced any AE of grade ≥3 judged to be related to pasireotide, worsening of hormone-related symptoms from carcinoid syndrome, or complications of cancer requiring surgery or radiotherapy. Treatment was also discontinued if the pasireotide LAR dose was delayed for >7 days.

### Patients

Adult male and female patients aged ≥18 years with biopsy-proven primary or metastatic well-differentiated GEP NET, refractory to available SSAs (octreotide or lanreotide), were enrolled in this trial. All patients had histopathologic confirmation of the diagnosis, elevated levels of chromogranin A or serotonin (within the previous 6 months, if possible), and Karnofsky performance status ≥60. Patients who received radiolabeled SSA therapy within the past 6 months were excluded.

All patients provided written informed consent. The protocol was approved by the institutional review board or independent ethics committee at each participating clinical center, and the study was conducted in accordance with Good Clinical Practice and the Declaration of Helsinki.

### Assessments

Safety assessments consisted of recording all AEs. Blood chemistry, CBC, urinalysis, vital signs, physical condition, ECG, and body weight were checked regularly throughout the study. In addition, an interim safety analysis was performed when six patients had completed the first 6 weeks of treatment. According to this prespecified analysis, enrollment and treatment were to be stopped and a comprehensive review of all safety data was to be undertaken if two or more patients in any dose cohort experienced serious AEs (SAEs; grade 3 or 4) judged related to pasireotide LAR.

PK blood samples were collected at predose (0 h) and 2, 4, 6, 8, and 10 h after the first pasireotide LAR injection on day 0 (the same day as injection); this was followed by PK blood collection at 24 and 26 h on day 1 after the first injection. Additional PK blood samples were collected at 8:00 AM (a single collection) during visits on days 7, 14, 21, and 28 after the first LAR injection. For both second and third LAR dosing, PK blood samples were collected only at 8:00 AM on days 14 and 28 after injections. Because of the sparse PK sampling, PK parameters of interest included only pasireotide maximum concentration (*C*_max,day0–1,1st inj_) from day 0 to day 1 after the first injection, the trough-level concentration (*C*_trough_) on day 28 after the first, second, and third injections, and the accumulation ratio (AR = *C*_trough,d28,3rd inj_/*C*_trough,d28,1st inj_).

PD response was measured by quantitative assessment of daily bowel movements using the carcinoid disease symptom diary.

### Statistical analysis

The all-patients population consisted of all patients who received at least one pasireotide injection (LAR, subcutaneous, or both), and the safety population consisted of all patients who received at least one pasireotide LAR injection. The PK population consisted of all patients who received at least one dose of pasireotide LAR and had evaluable PK assessments. This set was used for summary statistics of pasireotide concentration and PK parameters. The PD population consisted of all patients who received at least one pasireotide LAR injection and had evaluable PD assessments.

## Results

### Demographics and disposition

**Fig. 1:**
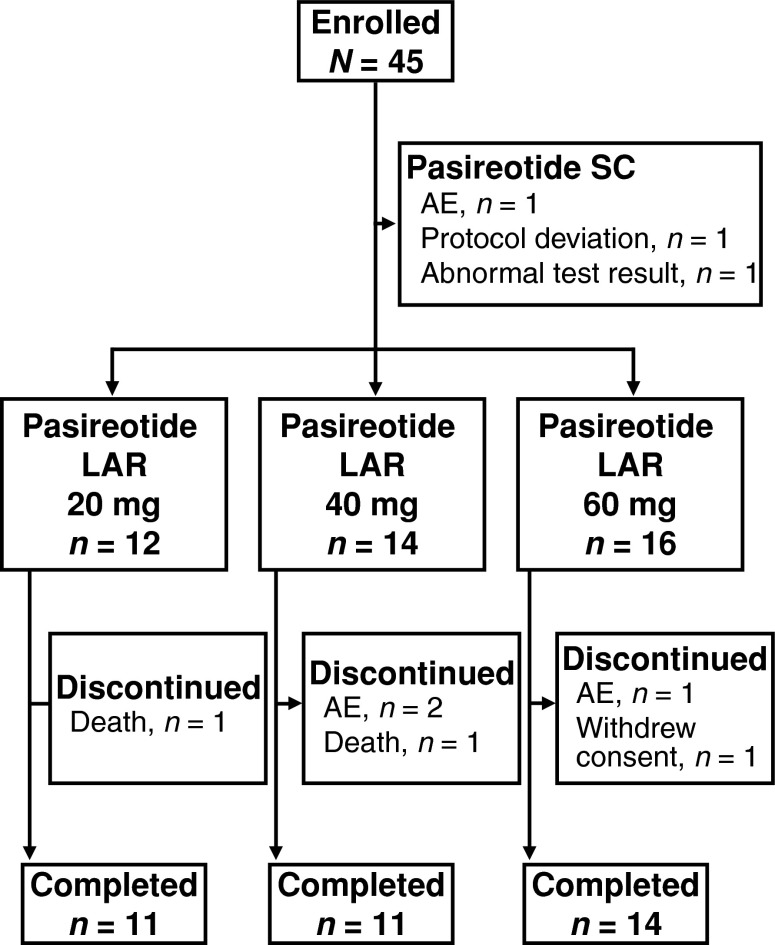
Patient disposition. *AE* Adverse event, *LAR* long-acting release, *SC* subcutaneous

In total, 45 patients were enrolled between June 2006 and September 2007; of those, 42 were randomly assigned to pasireotide LAR treatment (Table 1). Each of the three not randomly assigned received a single SC injection of pasireotide and was excluded from further participation in the study because of an AE (*n* = 1), a protocol deviation (*n* = 1), or an abnormal laboratory result (*n* = 1). All remaining 42 randomly assigned patients received the first dose of pasireotide LAR (20 mg, *n* = 12; 40 mg, *n* = 14; 60 mg, *n* = 16); 40 and 36 patients received the second and third doses, respectively (Fig. 1). Of the six patients who did not complete the study, three patients stopped because of an AE, two died of disease progression, and one withdrew consent. Patient demographics were comparable between treatment groups (Table 1).

**Table 1:** Patient demographics

	Pasireotide long-acting release			
	20 (mg) *n* = 12	40 (mg) *n* = 14	60 (mg) *n* = 16	Total *N* = 42
Mean age, years (SD)	56.3 (12.6)	58.9 (12.7)	62.5 (9.6)	59.5 (11.6)
Male/female, *n* (%)	9 (75)/3 (25)	6 (43)/8 (57)	7 (44)/9 (56)	22 (52)/20 (48)
Race, *n* (%)				
White	10 (83.3)	13 (92.9)	16 (100)	39 (92.9)
Black	1 (8.3)	0	0	1 (2.4)
Asian	1 (8.3)	0	0	1 (2.4)
Native American	0	1 (7.1)	0	1 (2.4)
Mean height, cm (SD)	176.4 (11.5)	169.5 (13.1)	168.9 (7.57)	171.2 (11.0)
Mean weight, kg (SD)	82.4 (15.9)	75.7 (18.7)	72.1 (12.3)	76.22 (15.9)
Mean BMI, kg/m^2^ (SD)	26.6 (5.3)	26.5 (6.7)	25.3 (4.0)	26.1 (5.3)
Median time since diagnosis, days (range)	1,403 (94–3,504)	1,372.5 (222–8,691)	1,054.5 (133–3,321)	1,238 (94–8,691)
Median time since most recent relapse, days (range)	117 (34–1,066)	288.5 (76–1,973)	226 (65–1,184)	182.5 (34–1,973)
Previous antineoplastic medications, *n* (%)				
Yes	7 (58.3)	9 (64.3)	13 (81.3)	31 (68.9)
No	5 (41.7)	5 (35.7)	3 (18.8)	14 (31.1)
Previous antineoplastic radiotherapy, *n* (%)				
Yes	0	0	1 (6.3)	1 (2.2)
No	12 (100)	14 (100)	15 (93.8)	44 (97.8)
Previous antineoplastic surgery, *n* (%)				
Yes	12 (100)	13 (92.9)	15 (93.8)	43 (95.6)
No	0	1 (7.1)	1 (6.3)	2 (4.4)

*BMI* Body mass index, *SD* standard deviation

### Safety and tolerability

Median duration of exposure was 84 days in all treatment arms. In total, 34 of 42 patients (81.0 %) receiving pasireotide LAR experienced at least one AE each. The most frequently reported were diarrhea (*n* = 12; 28.6 %), fatigue (*n* = 9; 21.4 %), abdominal pain (*n* = 8; 19.0 %), and nausea (*n* = 7; 16.7 %) (Table 2). No relationship was noted between incidence of AEs and dose of pasireotide. Treatment-related AEs were reported in 24 patients (57 %) at all dose levels. Diabetes mellitus (*n* = 5; 11.9 %) and hyperglycemia (*n* = 4; 9.5 %) were the only treatment-related AEs reported by more than two patients across all treatment groups.

**Table 2:** Summary of adverse events

	Pasireotide long-acting release			
Adverse events *n* (%)	20 (mg) *n* = 12	40 (mg) *n* = 14	60 (mg) *n* = 16	Total *N* = 42
Any adverse event	10 (83.3)	11 (78.6)	13 (81.3)	34 (81.0)
Diarrhea	4 (33.3)	4 (28.6)	4 (25.0)	12 (28.6)
Fatigue	3 (25.0)	3 (21.4)	3 (18.8)	9 (21.4)
Abdominal pain	3 (25.0)	4 (28.6)	1 (6.3)	8 (19.0)
Nausea	2 (16.7)	3 (21.4)	2 (12.5)	7 (16.7)
Diabetes mellitus	1 (8.3)	3 (21.4)	2 (12.5)	6 (14.3)
Dyspnea	2 (16.7)	3 (21.4)	1 (6.3)	6 (14.3)
Flushing	3 (25.0)	1 (7.1)	2 (12.5)	6 (14.3)
Headache	1 (8.3)	2 (14.3)	3 (18.8)	6 (14.3)
Anorexia	1 (8.3)	3 (21.4)	1 (6.3)	5 (11.9)
Asthenia	2 (16.7)	2 (14.3)	1 (6.3)	5 (11.9)
Treatment-related adverse event	6 (50)	10 (71.4)	8 (50)	24 (57.1)
Diabetes mellitus	1 (8.3)	3 (21.4)	1 (6.3)	5 (11.9)
Hyperglycemia	0	2 (14.3)	2 (12.5)	4 (9.5)
Serious adverse event	1 (8.3)	2 (14.3)	2 (12.5)	5 (11.9)
Discontinued because of adverse event	1 (8.3)	3 (21.4)	1 (6.3)	5 (11.9)

Individual AEs occurring in >10 % of patients across all treatment arms are presented

Fifteen patients reported grade 3/4 AEs (grade 3, *n* = 12; grade 4, *n* = 3). The most frequently reported grade 3/4 AEs (any dose) were diabetes mellitus and flushing (*n* = 3 each) and abdominal pain, increased glucose, hyperglycemia, hypokalemia, and liver metastases (*n* = 2 each); all other grade 3/4 AEs were individual reports of single events. Five patients discontinued treatment early because of AEs (20 mg, *n* = 1; 40 mg, *n* = 3; 60 mg, *n* = 1). In addition to the two patients who died, three patients discontinued treatment early because of nonserious AEs (abdominal pain, hyperglycemia, and diabetes mellitus).

Five patients had SAEs—the two who died and the three who had SAEs that did not lead to permanent study drug discontinuation. One patient in the 40-mg group had a small intestinal obstruction unrelated to study medication, and one patient in the 60-mg group had two SAEs of hyponatremia and liver metastases also unrelated to study medication. In addition, one patient in the 60-mg group had diabetes mellitus related to study medication, complicated by nonketotic hyperosmolar syndrome.

Two patients died during the study. One patient receiving pasireotide LAR 20 mg died of cancer progression 25 days after receiving the first dose of pasireotide. A second patient in the 40-mg group died 27 days after receiving the first dose of pasireotide. The cause of death was first reported as respiratory failure but was subsequently corrected by the investigator to cancer progression. In addition, one patient who received only subcutaneous pasireotide, but not the LAR formulation, died of cancer progression complicated by spinal cord compression more than 30 days after study discontinuation. No deaths were considered by investigators to be related to study drug.

### Laboratory values

Mean (median) fasting glucose levels were consistently increased compared with baseline at all points throughout the study. Baseline fasting blood glucose levels were 98.4 (96.3), 100.5 (95.8), and 98.2 (95.5) mg/dL, respectively, in the 20-, 40-, and 60-mg groups and increased to 126.4 (121.6), 129.4 (123.4), and 130.1 (125.0) mg/dL 2 days after the first dose of pasireotide. Levels remained elevated during the study and were 129.3 (115.0), 165.3 (130.6), and 160.8 (150.0) mg/dL, respectively, at the end of the study. Fasting insulin was slightly reduced in all treatment arms compared with baseline, but post-baseline it remained within the range of 4.2–7.4 mU/L in all treatment groups for the duration of the study. Mean (median) baseline fasting insulin level was higher in the 60-mg group at baseline than in the 20- and 40-mg groups (12.5 [8.5] mU/L vs 7.4 [6.7] mU/L and 7.2 [7.0] mU/L, respectively) but normalized by day 2 and remained within a clinically acceptable range for the remainder of the study. Overall, no effect of treatment on fasting blood glucagon levels was noted. At baseline, mean (median) glucagon levels appeared slightly higher in the 60-mg group compared with the 20- and 40-mg groups (82.3 [70.0] ng/L vs 72.8 [62.0] ng/L and 70.0 [59.5] ng/L), but in all dose groups, glucagon levels remained within the clinically accepted range for the whole study. Mean glycosylated hemoglobin (HbA_1c_) levels at baseline were 5.9, 6.0, and 5.9 % in the 20-, 40-, and 60-mg groups, respectively, and mean increases from baseline to study conclusion were 0.6, 1.2, and 1.4 %, respectively. Six patients developed detectable urine glucose during treatment.

Two patients in the pasireotide LAR 60-mg group had grade 3 hematologic abnormalities (decreased absolute lymphocytes, *n* = 1; decreased absolute neutrophils, *n* = 1); the only grade ≥3 laboratory abnormalities occurring in two or more patients across all treatment arms were total bilirubin (*n* = 2) and total triglycerides (*n* = 2). No significant changes in ECG were observed, and five patients had new or worsened gallbladder abnormalities at the end of the study.

### Pharmacokinetics

**Fig. 2:**
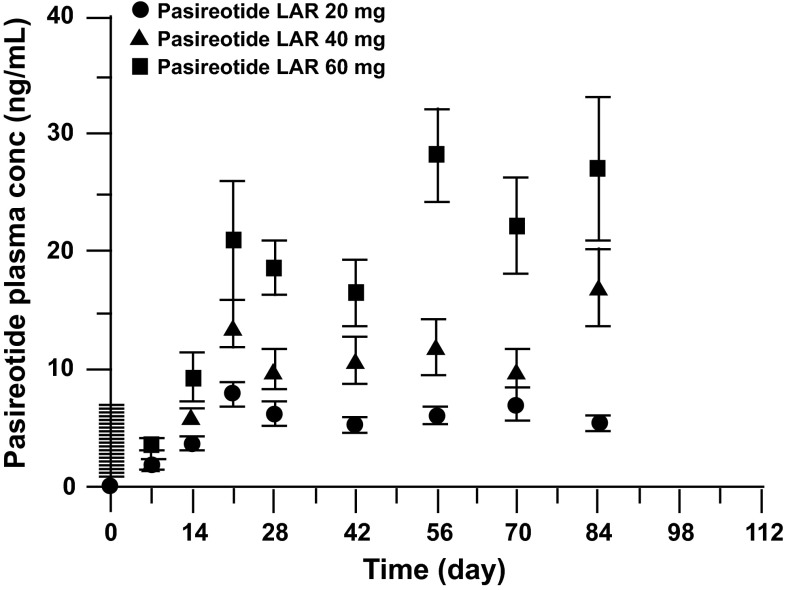
Plasma concentration versus time profiles of pasireotide in patients with gastroenteropancreatic neuroendocrine tumor (GEP NET) after three monthly injections. *LAR* Long-acting release

All 42 patients who received at least one dose of pasireotide LAR were evaluable for PK analyses. Mean (±SD) pasireotide plasma concentration vs time profiles for the three pasireotide LAR dose levels are shown in Fig. 2. Steady state levels of pasireotide were achieved in all treatment groups after the third LAR injection based on trough concentrations collected on day 28 after each dose (Fig. 2; Table 3). After the first injection of pasireotide LAR, an initial burst was noted during the first 24 h; median (mean ± SD) values of maximum plasma concentrations (*C*_max,d0-1,1st inj_) of pasireotide were 3.55 (4.50 ± 2.88), 5.39 (6.17 ± 4.08), and 6.09 (6.99 ± 4.09) ng/mL in the 20-, 40-, and 60-mg treatment arms, respectively (Table 3). Trough plasma concentrations of pasireotide on day 28 (*C*_trough,d28,1st inj_, median [mean ± SD]) after the first dose were 6.43 (6.31 ± 2.98), 8.56 (9.65 ± 5.89), and 16.5 (18.7 ± 9.3) ng/mL, respectively (Table 3). Trough concentrations after the third injection (*C*_trough,day28,3rd inj_, median [mean ± SD]) were 4.82 (5.6 ± 2.01), 12.0 (16.5 ± 10.2), and 19.7 (25.0 ± 20.5) ng/mL for the 20-, 40-, and 60-mg dose levels. These results suggested that PK exposures of pasireotide in patients with carcinoid disease had large variability and appeared to be slightly over dose proportional. However, because of the limited sample size of patients and the large interpatient variability in PK exposures, a definitive conclusion on dose proportionality could not be drawn. Median ARs were 0.998, 1.08, and 0.801, respectively, for the 20-, 40-, and 60-mg groups. In addition, concentration–time profiles from individual patients (data not shown) demonstrated that trough concentrations of pasireotide reached steady state after three LAR injections in most of the carcinoid patients.

**Table 3:** Pharmacokinetic parameters of pasireotide following monthly injection with LAR 20, 40, or 60 mg in patients with GEP NET

	Pasireotide long-acting release		
Pharmacokinetic parameters	20 (mg) *n* = 12	40 (mg) *n* = 14	60 (mg) *n* = 16
*C*_max,d0-1,1st inj_ (ng/mL)	3.55 (4.50 ± 2.88)	5.39 (6.17 ± 4.08)	6.09 (6.99 ± 4.09)
*C*_trough,d28,1st inj_ (ng/mL)	6.43 (6.31 ± 2.98)	8.56 (9.65 ± 5.89)	16.5 (18.7 ± 9.3)
*C*_trough,d28,2nd inj_ (ng/mL)	5.74 (6.14 ± 2.47)	9.68 (11.7 ± 7.7)	24.4 (27.6 ± 14.6)
*C*_trough,d28,3rd inj_ (ng/mL)	4.82 (5.5 ± 2.01)	12.0 (16.5 ± 10.2)	19.7 (25.0 ± 20.5)
AR (*C*_trough,d28,3rd inj_/*C*_trough,d28,1st inj_)	0.998 (1.05 ± 0.57)	1.08 (1.68 ± 1.14)	0.801 (0.896 ± 0.432)

Data are expressed as median (mean ± SD)

*AR* Accumulation ratio, *C*_max_ maximum concentration, *C*_trough_ trough-level concentration

### Pharmacodynamics

**Fig. 3:**
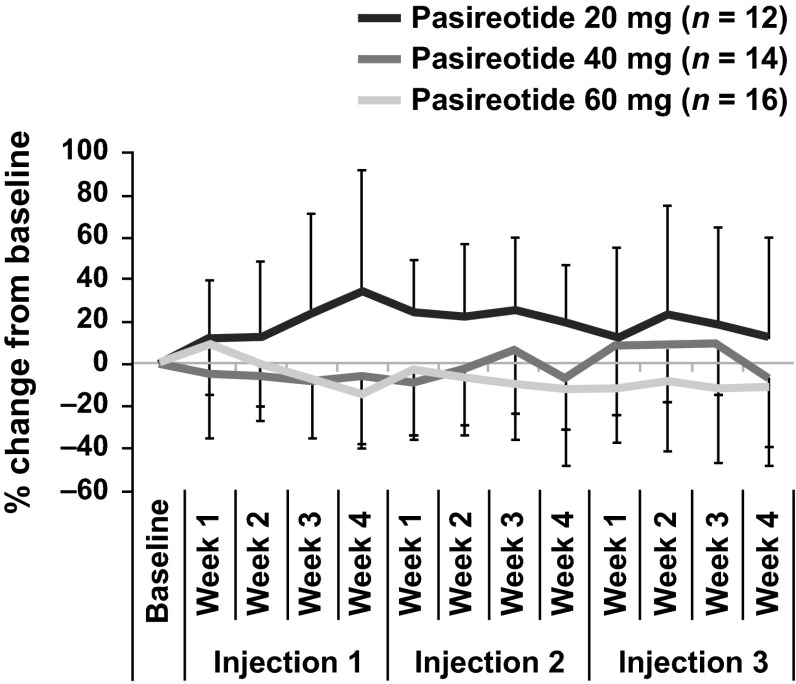
Percentage change from baseline in mean weekly bowel movements (data are mean ± SD)

The mean percentage change in average number of daily bowel movements per week showed a decrease from baseline at most time points for the 60-mg dose level, whereas for the 40-mg dose level, fluctuation relative to baseline levels was greater, and for the 20-mg dose group, an increase was reported at all post-baseline time points (Fig. 3). However, variability was high and sample size was limited.

## Discussion

SSAs such as octreotide represent the cornerstone of symptomatic therapy for patients with carcinoid syndrome from GEP NET; however, some patients develop resistance to these drugs, possibly mediated through adaptive processes at the cellular level [19–21]. These agents have high affinity for the sst_2_ receptor and modest affinity for the sst_5_ receptor, and uncoupling or downregulation of the sst_2_ receptor may be a possible explanation for the decline in efficacy occasionally seen with these drugs [19–22]. Pasireotide is a multireceptor-targeted agent with high affinity for four of the five known somatostatin receptor subtypes (sst_1–3,5_) and higher binding affinity than octreotide at sst_1_, sst_3_, and sst_5_ [12, 23]. As a result of its broader affinity for the somatostatin receptor subtypes [24], pasireotide may offer symptom control for patients resistant to medical treatment with octreotide or lanreotide.

Pasireotide LAR was well tolerated, with a safety profile consistent with that in previous reports [15]. Most AEs were mild or moderate and did not require pasireotide dose adjustment, consistent with findings of a previous study of subcutaneous pasireotide in patients with metastatic NET, in whom nausea, abdominal pain, weight decrease, and hyperglycemia were the most frequently reported (any grade) AEs [16]. During treatment, an increase in mean fasting blood glucose was noted, particularly in the 40- and 60-mg dose groups; however, insulin levels remained within normal ranges for the duration of the study. It is unclear why mean baseline insulin was elevated in the 60-mg dose group; the standard deviation for this group suggests substantial interpatient variability, as evidenced by the range of values (1–42 mU/L) and the relatively small number of subjects (*n* = 16). Glucagon levels also remained within the clinically acceptable range for the duration of the study, and HbA_1c_ levels at the end of the 3-month study were similar in all dose groups, suggesting adequate glucose control. Although not apparent in the present study, previous reports have suggested an association between long-acting formulations of SSAs and reduction in fasting plasma insulin but no significant effect on fasting plasma glucose [25]. Petersenn and colleagues [17] noted an effect of pasireotide on fasting blood glucose levels and suggested that it might have resulted from an effect on hepatic glucose production caused by the more pronounced suppression of insulin compared with glucagon.

Pasireotide LAR injected IM once every 28 days led to steady state pasireotide levels in all dose groups within three injections. An initial burst was observed during the first 24 h after the first LAR injection; the concentration then decreased to the lowest level around day 7, followed by an increase to the maximum concentration on day 21 after the first dose, reflecting the unique release profile of the pasireotide LAR formulation. The PK release pattern after the first dose in patients with GEP NET was similar to that in healthy volunteers [17], but PK exposures in patients with GEP NET were approximately twofold those in healthy volunteers. This twofold PK exposure difference between patients with GEP NET and healthy volunteers was also observed for the pasireotide SC formulation [16]. The underlying reason for the twofold PK exposure difference between these populations remains unknown.

It should be noted that because of the limitations of this study, including that it was not powered for efficacy endpoints, and that patient symptoms (including bowel movement frequency and flushing) were not controlled at baseline, no conclusions can be drawn regarding the control of diarrhea and flushing. Therefore, a randomized phase III study (Clinical Trial number NCT00690430) conducted to compare the efficacy of pasireotide LAR 60 mg IM every 28 days vs octreotide LAR 40 mg IM every 28 days is ongoing in patients with metastatic midgut NET whose disease-related symptoms are inadequately controlled by conventional doses of SSAs. It is anticipated that the results of this ongoing phase III study may provide new insight into the effects of pasireotide LAR on symptom control of diarrhea and flushing in carcinoid syndrome [16].

A recent systematic review of SSAs in the treatment of patients with GEP NET indicates that LAR formulations of octreotide (Sandostatin LAR; Novartis) and lanreotide (Somatuline SR/Autogel; Ipsen) provide symptom relief in 74.2 and 67.5 % of patients, and that 69.8 and 64.4 %, respectively, experience tumor control [8]. The ability of SSAs to control tumor growth was demonstrated in the phase III PROMID study, in which octreotide LAR significantly prolonged time to tumor progression compared with placebo in patients with metastatic midgut NET (14.3 vs 6 months; hazard ratio 0.34; 95 % confidence interval 0.20–0.59; *P* = 0.000072) [26]. Overall, 66.7 % of patients receiving octreotide and 37.2 % of those receiving placebo had stable disease after 6 months of therapy [26]. In the phase III RADIANT-2 study, the addition of everolimus to octreotide suggested an extension of progression-free survival [27].

A number of elements regarding the design of this study are worth additional comment. Recently released consensus guidelines establish key unmet needs, develop appropriate study endpoints, and standardize clinical trial inclusion criteria for research on NET [28]. Relevant to the present study, these guidelines advocate avoidance of a washout period before study initiation as ethically unacceptable and unnecessary provided study durations are sufficiently long to rule out carryover effects from previous medications. The present study was completed before publication of these guidelines; hence, we could not consider this recommendation during study design. However, with the potential for saturation of pasireotide elimination pathways at higher doses, it is equally possible that the concurrent presence of any agents using the same elimination pathways (e.g., carryover of a previously used long-acting SSA) could result in artificially increased pasireotide plasma concentrations during the early stages of the study.

In conclusion, a new once-monthly IM LAR formulation of pasireotide was well tolerated in patients with GEP NET refractory to available SSAs, with a toxicity profile consistent with that in previous reports. Steady state levels of plasma pasireotide were achieved within three injections. These observations support the continued evaluation of pasireotide LAR in patients with GEP NET. An ongoing study is being conducted to determine the maximum tolerated dose above 60 mg, and a comparative randomized phase III study is expected to provide further data regarding the clinical effectiveness of this treatment. Moreover, the tolerability profile of pasireotide supports the potential use of this new agent in combination regimens.
